# Redox Mechanisms Underlying the Cytostatic Effects of Boric Acid on Cancer Cells—An Issue Still Open

**DOI:** 10.3390/antiox12061302

**Published:** 2023-06-19

**Authors:** Giulia Paties Montagner, Silvia Dominici, Simona Piaggi, Alfonso Pompella, Alessandro Corti

**Affiliations:** Department of Translational Research NTMS, University of Pisa Medical School, 56126 Pisa, Italy; g.patiesmontagner@studenti.unipi.it (G.P.M.); silvia.dominici@unipi.it (S.D.); simona.piaggi@unipi.it (S.P.); alfonso.pompella@unipi.it (A.P.)

**Keywords:** boric acid, boron, cancer, apoptosis, ferroptosis

## Abstract

Boric acid (BA) is the dominant form of boron in plasma, playing a role in different physiological mechanisms such as cell replication. Toxic effects have been reported, both for high doses of boron and its deficiency. Contrasting results were, however, reported about the cytotoxicity of pharmacological BA concentrations on cancer cells. The aim of this review is to briefly summarize the main findings in the field ranging from the proposed mechanisms of BA uptake and actions to its effects on cancer cells.

## 1. Introduction

Boron is a non-metallic element existing in nature as borax (sodium tetraborate; Bx) and boric acid (BA), the latter being the dominant form of boron in plasma. Boron is abundant in foods, particularly in fruits and seeds, and its levels in animals are tightly controlled by homeostatic mechanisms that primarily involve renal excretion. BA is very well absorbed following oral administration, is not metabolized and is mainly excreted in urine. Boron concentration has been reported to be about 10–20 µM in human plasma, whereas in tissues, the highest concentrations were reported for bone, heart, spleen and liver [1]. On the other hand, upon exposure to high doses or as a consequence of boron deficiency, toxic effects have been reported in animals as well as humans, including death.

Several studies have been reported focusing on the physiological functions of boron (e.g., see [2]), which is known to be important for animal cell replication and development. In addition, some studies also suggested that BA could be preventive and have therapeutic effects in a number of cancers (e.g., [3,4,5,6]), although different and—in some cases—contrasting results were reported (e.g., [7,8,9,10]).

The aim of this short review is to critically summarize the main findings in the field.

## 2. Physico-Chemical Properties of BA and Cellular Uptake

The pKa of BA ranges around 9–9.2, depending on temperature, ionic strength and concentration used [11]. At physiological pH, BA may enter the cells through a ubiquitously expressed Na^+^-coupled borate transporter (NaBC1) and dissociates as borate anion plus H^+^, thus decreasing the intracellular pH. In the absence of borate, NaBC1 transports Na^+^ and OH^−^ (H^+^), while in the presence of borate, NaBC1 functions as an electrogenic, voltage-regulated, Na^+^-coupled B(OH)_4_^−^ transporter. The levels of expression of NaBC1 could thus modulate the final intracellular concentrations of borate [12,13]. On the other hand, the role of human aquaporins in BA uptake was also proposed [14], whereas in a study on human hepatoma HepG2 cells, the primary mechanism proposed for BA uptake was a simple diffusion, with some factors, such as low pH and lipid composition of cells membranes, modulating the membrane permeability to BA [15].

At intracellular pH (7.4), BA is a mild organic Lewis acid and—like boronic acid compounds as well—it can form complexes with Lewis bases such as hydroxide anions and electron-donating groups (such as nitrogen or oxygen), thus behaving as an electrophile [11]. Boron is immediately to the left of carbon in the periodic table and may form small compounds of a size appropriate for targeting key binding sites, just as carbon does [16], making it an alternative to carbon in drug design [17].

BA and boronic acids can interact with functional groups such as alcohols, carboxylic acids, thiols and amines, allowing the formation of reversible non-ionic bonds with enzyme residues, nucleic acids or hydroxyl groups from carbohydrates [11,18]. Boron compounds may thus potentially interact with different metabolites and enzymes, thus influencing cellular activities. Indeed, it was proposed that boron may influence the activity of enzymes in different ways, i.e., by directly binding to cofactors or substrates, or by unknown mechanisms [19]. Early studies suggested that boron compounds may bind to the active site of serine proteases to form a reversible transition state analogue complex [20]. This feature would induce a boron-dependent inhibition of various enzymes, such as the prostate-specific antigen (PSA), histone deacetylases (HDAC) and oxidoreductases (e.g., [19,21,22,23]). On this basis, various boron-derived compounds have been used for the development of enzyme inhibitors of proteases—including proteasoma—arginase, nitric oxide synthase and transpeptidases [18].

**Figure 1:**
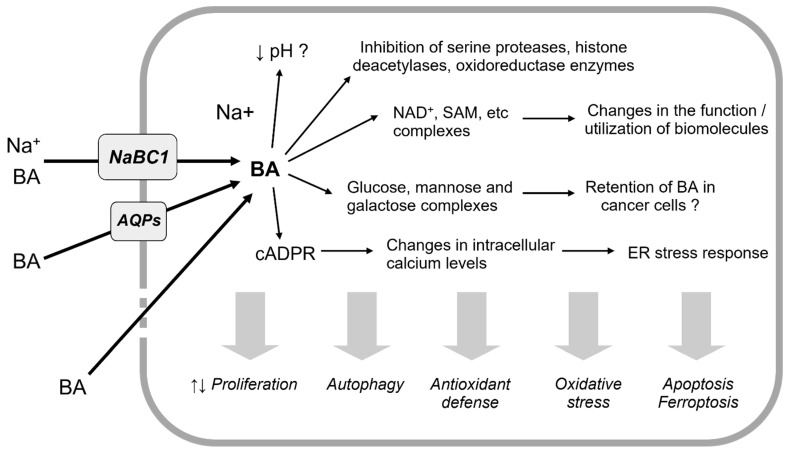
Overview of the proposed mechanisms of action of BA. The mechanisms underlying the apparent dichotomous functions of BA and—more importantly—the possibility of reaching suitably high concentrations of BA in the tumor microenvironment need to be deeply investigated.

Early studies showed that several dehydrogenases, including alcohol, lactate, phosphogluconate, glucose-6-phosphate, glyceraldehyde-3-phosphate and succinate dehydrogenases, may also be competitively inhibited by borate [18,24]. These effects can be explained by a competition between borate and enzymes for the substrate NAD^+^. Indeed, BA is able to bind to molecules with vicinal *cis*-diols groups, with a high affinity for the ribose moieties of NAD^+^ [25] and for S-adenosylmethione [26]. In particular, *cis*-1,2-diols are favored over trans- or 1,3-diols, and five-membered ring 1,2-diols are preferred over six-membered ring 1,2-diols [27]. Moreover, the binding affinity of borate significantly depends on the charge and phosphorylation status of substrates, with an inverse and proportional correlation between the number of nucleotide phosphate groups and the relative abundance of the borate complexes [28]. BA can form complexes with *cis*-diols containing carbohydrates as well, e.g., glucose, mannose and galactose, with this possibly modulating the accumulation of BA inside cells [15] (Figure 1).

## 3. Effects of Boric Acid on Cancer Cells: In Vivo and In Vitro Studies

The peculiar physico-chemical properties of BA (and its derivatives) on one side, and some epidemiological studies correlating a reduction in prostate cancer risk with BA dietary intake on the other (e.g., [3,4]), have prompted some authors to study the effects of BA on cancer cell viability and proliferation.

This is quite a complex field with much of the current knowledge about the mechanism of action of BA in cancer cells coming from in vitro studies. The major differences in these studies come from the (largely) different BA concentrations used (physiological vs. pharmacological), the time of incubation and the model used, with all possibly contributing to the—sometimes antithetical—effects observed.

### 3.1. In Vivo Studies

Some in vivo studies focused on the effects of BA administration on the progression of tumor xenografts. Treatments with BA (1.7–9.0 mg/kg/day) of nude mice implanted subcutaneously with human prostate adenocarcinoma LNCaP cells showed decreased cell proliferation, tumor size, prostate-specific antigen (PSA) levels and intratumoral expression of IGF-1 [29]. In another study on rat hepatocellular carcinoma (HCC), borax (4 mg/kg/day; Bx) treatment reduced Proliferating Cell Nuclear Antigen (PCNA) expression and [3H]-thymidine incorporation as markers of cell proliferation [30]. Bx treatment also counteracted the modifications of biochemical markers of hepatic injury and oxidative stress associated with cancerous transformation, thus reducing lipid peroxidation and the activity of glutathione transferase, molybdenum Fe–S containing flavin hydroxylases and glucose 6-phosphate dehydrogenase. On the other hand, boron treatment also increased the levels of the major antioxidant glutathione and the activities of glutathione reductase, glutathione peroxidase, catalase and superoxide dismutase enzymes [30].

Other studies focused on the combination of BA with chemotherapeutic drugs. The alkylating agent cyclophosphamide is a drug used in cancer treatment, causing cellular oxidative and nitrosative stress with severe side effects on heart, kidney, liver, bladder and bone marrow [31]. Indeed, BA (200 mg/kg/day) was able to protect rat tissues against cyclophosphamide-induced bladder [32] and testicular [33] damage, thanks to its antioxidant and anti-apoptotic effects. Cisplatin (*cis*-diaminodichloroplatinium; CDDP) is another alkylating agent used in cancer treatment causing oxidative stress with primary side effects on kidneys. In a study investigating CDDP nephrotoxicity in rats, different doses of BA (50–200 mg/kg) produced different effects on the drug-induced markers of oxidative and ER-related stress [34]. It can be envisaged that major differences in the experimental conditions used, including the animal model (mouse, rat), the route of BA administration (oral, injection), the duration of treatment (days, months) and the different boron compounds (BA, Bx), may account for the differences observed in these studies. Moreover, boron is quickly excreted in the urine of both animals and humans [35]. All these factors make it difficult to establish comparisons with data obtained from “static” in vitro models.

### 3.2. In Vitro Studies

A large number of studies regarding the effects of BA focused on cellular models of prostate cancer. DU-145 prostate cancer cells were largely used for investigating cellular processes modulated by BA because their proliferation is sensitive to BA over a wide concentration range [36].

In vitro studies can be grouped considering the levels of BA used, i.e., pharmacologically (high micromolar/millimolar) or physiologically (low micromolar) relevant concentrations (Table 1).

As far as *pharmacologically relevant concentrations*, BA reduced the proliferation, migration and invasion of human cancer prostate cell lines in vitro in a dose-dependent manner, over a range from 0.06 to 1 mM [4,36]. Studies on DU-145 prostate cancer cells showed that BA was able to induce the cell-death-independent inhibition of proliferation, with little effect on cell cycle stage distribution and mitochondrial function. Moreover, non-tumorigenic prostate cells RWPE-1 and PWR-1E displayed reduced sensitivity towards BA as compared with cancer cell lines DU-145 and LNCaP, with this suggesting that the selected cellular mechanism(s) might modulate BA sensitivity [36]. In this perspective, a different efficiency in borate transport was also proposed [13,15]. In a further study on DU145 prostate cancer cells, a prolonged (8 days) exposure to 1 mM BA was shown to induce a conversion to a senescent-like cellular phenotype, a dose-dependent reduction in cyclins A–E and p21 and a reduced phosphorylation of MEK (P-MEK1/2) and ERK (P-ERK1/2) [4]. These modifications resulted in reduced cell motility, invasion capacity and proliferation [4], with the latter effect possibly supported by the formation of nucleotide–borate complexes altering nucleotides utilization by cells [36].

In accordance with these results, a cell-death-independent proliferative inhibition by millimolar concentrations of BA (0.5–20 mM) was also described for the human breast cancer cell line MDA-MB-231 after only 24 h of incubation. No significant effects on Bcl-2 protein levels or cytochrome c release were detectable, and only minor modifications were observed for caspase-3 activity [37].

At variance, other studies identified a possible connection between BA effects and the induction of apoptosis. In a recent study on DU-145 cells, a short, 24 h, millimolar (6–16 mM) BA treatment was associated with significantly increased cytochrome c and caspase-3 levels, suggesting the induction of apoptotic cell death [10]. Similarly, 0.5–1 mM BA induced low levels of apoptosis in the acute leukemia cell line HL-60 after 24 h of incubation [38]. Finally, other studies on human skin melanoma cells (SK-MEL28 [39]), small-cell lung cancer cells (DMS-114 [40]), human colon adenocarcinoma cells (SW-480 [41]) and human hepatocellular carcinoma cells (HepG2 [42]; Mahlavu, HuH-7 [43]) also detected apoptosis induction upon exposure to higher millimolar (0–100 mM) concentrations of BA.

Part of these studies also pointed out that BA would be able to modulate the redox state of the cell, thus contributing to apoptotic cell death. A short (24 h) exposure of DU145 prostate cancer cells to millimolar concentrations of BA (6–16 mM) was able to reduce cell viability and induce oxidative stress by decreasing superoxide dismutase (SOD) and catalase (CAT) activities and the levels of intracellular glutathione. Accordingly, a significant increase in malondialdehyde (MDA) levels was also observed, with this suggesting that BA would exert major cytotoxic effects by reducing antioxidant levels [10].

Similar results were also obtained in a study on the human glioblastoma cell line U-87MG where a 24 h incubation with increasing concentrations of BA (from 0.02 to 13 mM) induced a reduction in cell viability and increased oxidative stress. MDA levels along with SOD and CAT enzyme activities were increased, whereas glutathione levels as well as proinflammatory cytokines (i.e., IL-1α, IL-6, TNF-α) appeared to be decreased. Finally, BRAF/MAPK, PTEN and PI3K/AKT signaling pathways were also modulated [44].

It can be envisaged that part of the discrepancies about the inhibitory effects on cell proliferation vs. apoptosis by BA may be explained by the different cellular models and/or by the BA concentrations used. Similarly, the changes described for parameters indicating oxidative damage (e.g., MDA, glutathione) and the levels of antioxidant defenses (e.g., SOD, CAT) might reflect a model of reversible cell injury/adaptation, with the initial consumption (or inactivation) of antioxidant defenses and the following induction of the antioxidant response. More damaging conditions would result in irreversible cell injury and apoptosis. In this perspective, a “bell-shaped” dose response for borate’s effect on cellular growth was demonstrated in both HEK293 and HeLa cells, and the modulation of differential MAPKs’ pathway was proposed to play a role. BA was shown to be mitogenic at concentrations ranging from 0.1 to 0.5 mM and to inhibit cell growth at concentrations above 1 mM [12].

In this perspective, additional useful information comes from in vitro studies with *physiologically relevant BA concentrations*. A study on DU-145 cells confirmed that a treatment with 10 μM BA was able to inhibit cell proliferation without inducing apoptosis. The same treatment was also able to activate eukaryotic initiation factor 2 (eIF2α) and the two ATF4- and ATF6-dependent pathways. The activation of eIF2α is implicated in cell response against endoplasmic reticulum stress [45]. The same group then proposed an intriguing mechanism of regulation by physiological levels of BA. BA is a reversible dose-dependent competitive inhibitor of cyclic ADP ribose (cADPR), the endogenous agonist of the ryanodine receptor that stimulates Ca^2+^ release [46]. The inhibition by BA of cADPR-stimulated Ca^2+^ release also results in decreased levels of internal ER Ca^2+^, possibly due to a subsequent, altered activation of STIM proteins that are involved in triggering Ca^2+^ influx into the ER. Lowered levels of ER Ca^2+^ concentrations activate protein kinase RNA-like endoplasmic reticulum kinase (PERK). Finally, PERK may phosphorylate and activate eIF2α as well as Nrf2 factors, resulting in the activation of the eIF2α/ATF4 and Nrf2/Keap-1 pathways [8,47]. Interestingly, some of the genes associated with ER stress were also found to be stimulated in rats treated with CDDP and BA [34].

Phosphorylation of eIF2α activates a transcriptional program allowing cells to adapt to stress, e.g., by stimulating the removal of unfolded proteins, by activating autophagy or apoptosis. The level of phosphorylated eIF2α addresses the cellular response towards adaptation/cytoprotection or apoptosis when the cells are irreversibly or chronically damaged [48]. On the other hand, Nrf2 is a transcription factor that regulates the expression of genes involved in the cellular defense against toxic and oxidative insults as well as in metabolism and inflammation [49]. Indeed, Nrf2 activation by BA modulates the expression of detoxifying enzymes such as γ-glutamylcysteine synthetase (GCLC) and NAD(P)H:quinone oxidoreductase 1 (NQO1) [8]. GCLC is a rate-limiting enzyme in glutathione biosynthesis [50], and its modulation by BA would help explain the increased levels of intracellular glutathione observed in some studies.

This protective—rather than damaging—modulatory effect of physiological BA concentrations would also help explain how it may protect against the oxidative damage induced by different chemicals (e.g., [51,52,53,54]) as well as how it may induce cell proliferation/inhibition with a “bell-shaped” dose response [12].

Again, cellular models and experimental conditions used may determine the final effects produced by BA. Indeed, in a study on MDAH-2774 ovarian cancer cells, the same treatment with 10 μM BA produced antiproliferative effects. Moreover, a significant increase in apoptosis-inducing genes (e.g., *BAX*, *BID*, *CASP-3* and *CASP-9*) and a significant decrease in negative regulators of apoptosis (e.g., *BCL-2* and *BCL-xL*) were associated with a significant increase in apoptosis. Further effects included the inhibition of cell migration and increased levels of oxidative stress [55].

**Table 1:** Experimental conditions and cell lines used in some in vitro studies with BA. (↑) Increase; (↓) decrease; (-) no change.

Cell Line	BA Concentration	Time of Incubation	Effects Observed	Reference
*Pharmacological BA concentrations*				
Human prostate cancer cell lines: DU145, LNCaP, PC-3 Human non-tumorigenic prostate cell lines: RWPE-1, PWR-1E	0.060–1 mM	8 days	↓ proliferation ↓ migration ↓ invasion - caspase-3 - DNA fragmentation	[36]
Human prostate cancer cell line DU145	0.5–1 mM	8 days	↓ proliferation ↓ adhesion ↓ migration ↓ invasion Senescent-like phenotype ↓ cyclins A–E ↓ p21 ↓ p-MEK1/2 ↓ p-ERK1/2	[4]
Human acute leukemia cell line HL-60	0.5–1 mM	24 h	↓ cell viability minor apoptosis increase	[38]
Human embryonic kidney cells HEK293 Human cervical cancer cells HeLa	0.1–10 mM	16 h	↑/↓ proliferation with a “bell-shaped” curve ↑ p-MEK1/2 ↑ p-ERK1/2	[12]
Human glioblastoma cell line U-87MG	0.02 to 13 mM	24 h	↓ cell viability ↑ MDA levels ↑ superoxide dismutase ↑ catalase ↓ glutathione ↓ IL-1α, IL-6, TNF-α ↓ BRAF/MAPK ↓ PTEN ↓ PI3K/AKT	[44]
Human prostate cancer cell line DU145	6–16 mM	24 h	↓ cell viability ↑ cytochrome C ↑ caspase-3 ↑ apoptosis ↑ oxidative stress ↑ MDA levels ↓ superoxide dismutase ↓ catalase ↓ intracellular glutathione	[10]
Human breast cancer cell line MDA-MB-231	0.5–20 mM	24 h	↓ proliferation minor modification of caspase-3 activity	[37]
Human hepatocellular carcinoma cell line HepG2	0.5–40 mM	24 h	↓ cell growth ↑ DNA damage ↑ apoptotic and senescence-like transcripts ↑ phase I/II metabolic enzymes	[42]
Human melanoma cell line SK-MEL28	0–50 mM	1–10 days	↓ proliferation ↑ apoptosis	[39]
Human small-cell lung cancer cell line DMS-114	0–60 mM	24–72 h	Cell cycle arrest (G2/M phase) ↑ apoptosis ↑ *BAX* ↑ *CASP3* ↓ *BCL-2*	[40]
Human colon adenocarcinoma cells SW-480	10–100 mM	24–72 h	↓ cell viability ↑ apoptosis	[41]
Hepatocellular carcinoma cell lines Mahlavu and HuH-7	1–160 mM	24–48 h	↓ cell viability ↓ migration ↑ apoptosis ↑ autophagy ↑ caspase 3 ↓ pAKT	[43]
*Physiological BA concentrations*				
Human prostate cancer cell line DU145	10 μM	2–3 h	↑ PERK ↑ eIF2α ↑ Nrf2 ↑ GCLC ↑ NQO1	[8]
Human prostate cancer cell line DU145	10 μM	0–24 h	↓ proliferation ↑ eIF2α ↑ ATF4 ↑ ATF6	[45]
Human ovarian cancer cell line MDAH-2774	10–50 μM	24 h	↓ proliferation ↑ apoptosis ↓ cell migration ↑ oxidative stress ↑ *BAXx* ↑ *BID* ↑ *CASP3* ↑ *CASP9* ↓ *BCL-2* ↓ BCL-xL	[55]

## 4. BA-Dependent Redox Effects: A Still Provisional Summary

The picture that emerges from in vitro studies thus suggests that boric acid could support cell proliferation at micromolar concentrations while inhibiting cell growth beyond a millimolar threshold. The knowledge about the molecular mechanisms supporting the protective or toxic effects of BA is still limited, but the pathways that have been identified are mostly supposed to be activated by BA’s ability to form complexes with key biomolecules, including a large number of enzymes. This seems to be the background, e.g., for the effect of micromolar BA concentration on ryanodine receptor, leading to PERK activation [8] as well as for the effects of millimolar BA concentrations on histone deacetylase inhibition, possibly contributing to DNA damage [42].

**Figure 2:**
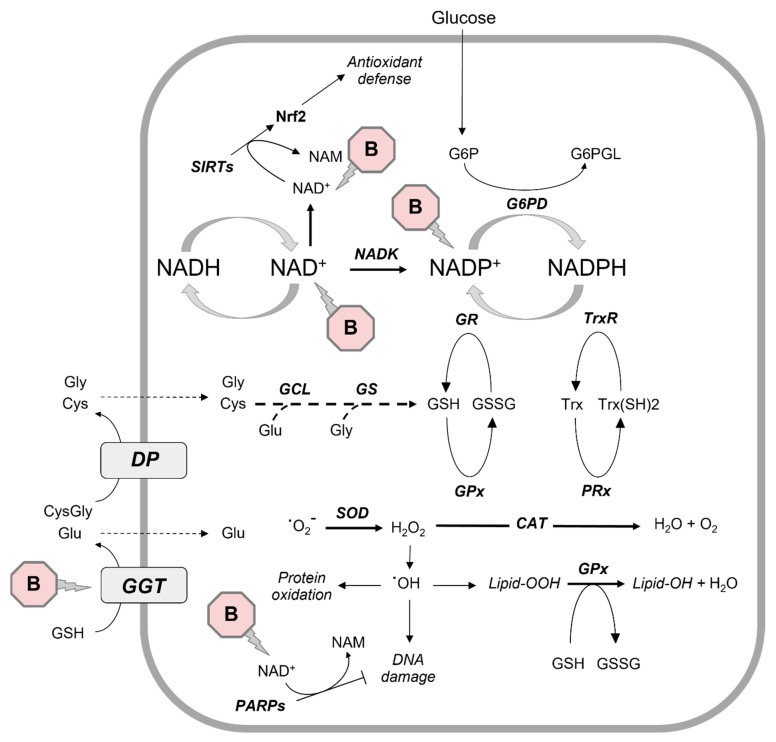
Overview of the proposed effects of boron on NAD^+^ and possible consequences on cellular antioxidant defenses. B, boron; CAT, catalase; Cys, cysteine; CysGly, cysteinylglycine; DP, dipeptidase; GCL, glutamate cysteine ligase; GGT, gamma-glutamyl trasnferase; GS, glutathione synthetase; Glu, glutamate; Gly, glycine; GPx, glutathione peroxidase; GR, glutathione reductase; GSH, reduced glutathione; GSSG, glutathione disulfide; G6P, glucose 6−phosphate; G6PGL, glucono 1,5−lactone 6−phosphate; G6PD, glucose−6−phosphate dehydrogenase; Lipid−OOH, lipid peroxide; Lipid−OH, lipid alcohol; NAD^+^, nicotinamide adenine dinucleotide (oxidized form); NADH, nicotinamide adenine dinucleotide (reduced form); NADK, nicotinamide adenine dinucleotide kinase; NADP^+^, nicotinamide adenine dinucleotide phosphate (oxidized form); NADPH, nicotinamide adenine dinucleotide phosphate (reduced form); NAM, nicotinamide; Nrf2, nuclear factor erythroid 2−related factor 2; PARPs, poly (adenosine diphosphate-ribose) polymerases; PRx, peroxiredoxin; SIRTs, sirtuins; Trx, thioredoxin; TrxR, thioredoxin reductase; Trx(SH)2, thioredoxin reduced form.

The increased oxidative stress parameters—where observed—could therefore reflect the inactivation of antioxidant enzymes or a possible “direct” effect of boric acid, as suggested by others. As regards the major intracellular antioxidant glutathione, it has been long known that the serine–borate complex is an inhibitor of gamma-glutamyl transferase (GGT, [56]), a critical enzyme implicated in glutathione metabolism. Borate may also modulate the activity of several dehydrogenases [25], including glucose-6-phosphate-dehydrogenase, playing a key role in the maintenance of the nicotinamide–adenine dinucleotide phosphate (NADPH) needed to keep glutathione and thioredoxin in their reduced forms [57,58]. In this perspective, it was demonstrated that borate may interact with *cis*-diol groups of nicotinamide nucleotides, with a binding affinity significantly depending on the charge and phosphorylation status of substrates (NAD^+^ > NADH > NADP^+^ > NADPH) [25,28]. Indeed, it was suggested that the borate–NAD^+^ complex may be the most physiologically relevant one [28]. It cannot be excluded, however, that—in those studies using high millimolar concentrations of BA in vitro—borate might interfere with NADP^+^ production from NAD^+^ [59] and that other less physiologically relevant complexes (e.g., borate–NADP^+^) may also be formed (Figure 2). Moreover, some authors reported that BA may inhibit the NAD^+^ and NADP^+^-induced release of stored Ca^2+^ in DU-145 prostate cancer cells [60].

Boron was demonstrated to be an inhibitor of histone deacetylases (HDAC) as well [22]. Sirtuins are class III histone deacetylases, whose enzymatic activity is dependent on NAD^+^ as a cofactor. It was suggested that Sirtuin 1 may protect cells from oxidative stress by modulating nuclear accumulation, DNA binding and transcriptional activity of Nrf2 [61], thus favoring the expressions of Nrf2 downstream genes such as heme oxygenase-1, superoxide dismutase 1, catalase and—as stated above—GCLC, a rate-limiting enzyme in glutathione biosynthesis [50]. It can be speculated that high concentrations of BA may also interfere with the defense pathways supported by histone deacetylases. Indeed, high BA concentrations seem to induce—where measured—a decrease in intracellular glutathione and an increase in MDA, whereas data about superoxide dismutase and catalase activities are more contrasting [10,44]. The formation of the borate–NAD^+^ complex could also produce effects on the activity of Poly(ADP-ribose) polymerase 1 (PARP1), a DNA damage sensor whose enzymatic activity is rapidly activated in response to DNA damage. PARP1 is involved in multiple DNA repair pathways and uses oxidized NAD^+^ as a substrate to ADP(ribosyl)ate itself and various target proteins [62].

Finally, it was also hypothesized that toxicity of BA might result from the ability of high concentrations to impair Ca^2+^ signaling [46,60].

Early findings suggested that boron is a regulator of enzymatic activity involved in energy production by cells. It can be envisaged that boron may affect the activity of sensitive enzymes, resulting in mild oxidative stress and activation of an adaptative response (e.g., through Nrf2 activation) [8]. On the other hand, high doses of BA could more drastically inhibit the production of reducing power in cells, thus resulting in oxidative damage. In this perspective, BA itself would not be an antioxidant, but it could indirectly induce antioxidant defense (e.g., glutathione biosynthesis) at low/physiological concentrations [9], while preventing its regeneration at high doses.

Finally, it must be considered that the possible interference of boron with the energy metabolism could differentially impact on cancer cells, i.e., cells characterized by an impaired metabolism (Warburg effect) and diversified patterns of mutations affecting proto-oncogenes and tumor suppressor genes [63]. These effects could lead to greater alterations in the production of reducing power in tumor cells than in normal cells.

## 5. Optimization of Boron Delivery to Cancer Cells: Synthetic Boron Derivatives

As judged by in vitro data, cancer cells seem to be more sensitive to BA effects [36,38,40]. It was hypothesized that a differential expression of the NaBC1 transporter [13] or physical-chemical factors such as low pH of the acidic tumor microenvironment and lipid composition of cancer cell membranes—modulating cell membrane fluidity and channels permeability [15]—might modulate the uptake and the final intracellular concentrations of BA in cancer cells. BA can also form complexes with *cis*-diols containing carbohydrates, i.e., glucose, mannose and galactose, and it was also suggested that it could modulate the accumulation of BA in tumors by preventing its diffusion out of cancer cells [15]. Finally, considering that cancer cells may be more vulnerable to agents that impair redox balance and increase oxidative stress [64], high BA concentrations might be more damaging for them, possibly through the modulation of energy production [19]. In this perspective, BA and boronates could thus be exploited as chemo-sensitizer agents in order to induce/enhance ferroptosis [65,66].

However, the possibility of reaching suitable high concentrations of BA in the tumor microenvironment may be another issue. It was observed that the therapeutic window of BA for prostate cancer cells is ∼100 times higher than its average serum level in humans, suggesting the difficulty in the systemic administration of soluble B compounds such as BA without toxicity. Moreover, it was also suggested that systemic administration of soluble boron compounds, such as BA, may be hampered by their short half-life, low bioavailability, requirement of frequent administration, low fraction arriving in the tumor site and limited effectiveness [67]. Indeed, the half-life of BA in humans is in the order of 24 h and is quickly excreted via urine [35].

Similarly, the mentioned “dual effect” of BA should also be taken into account when considering its possible use in combination with chemotherapeutics. Indeed, BA seems to prevent the genotoxicity of paclitaxel on human lymphocytes [52], and a pre-treatment with BA protected bladder [32] and rat testis [33] against cyclophosphamide-induced damage.

However, the unique chemical properties of boron have led to the development of numerous derivatives (i.e., boronic acids) for different applications [18]. Based on their electronic structures, boronic acid compounds have been studied for their potential use in the development of enzyme inhibitors or feedback-controlled drug release systems [68]. Some authors suggested that sugar–borate complexes, i.e., mono- or di-sugar–borate esters produced by plants, comprising one or two monosaccharide molecules linked to a boron atom (a primary natural dietary source of boron in humans [69]), could act as boron vehicles and increase borate levels inside cancer cells [70]. On the other hand, boron nitride (BN) spheres were also proposed as possible carriers. BN spheres have been used as a vehicle for the delivery of anticancer drugs such as doxorubicin [71], but hollow BN spheres with controlled crystallinity and boron release were also shown to be effective by itself in increasing apoptosis and necrosis in a mouse model of prostate cancer [67]. In this perspective, the administration of hollow BN spheres with a chemotherapeutic may have synergistic effects in the suppression of tumor growth.

Boron derivatives are also used in boron neutron capture therapy (BNCT). BNCT is a binary form of cancer radiation therapy aimed at improving the treatment of invasive malignant tumors such as glioblastoma multiforme, head and neck cancers, breast cancers and hepatocellular carcinoma, among others. Treatment begins with an injection of boron-containing drugs (e.g., boronophenylalanine (BPA), borocaptate sodium (BSH)) followed by thermal neutron irradiation. BPA mainly targets proliferating tumor cells, is transported by L-amino acid transporters [72] and produces higher boron concentration in tumor cells [73,74]. The BNCT therapy—along with the use of the boron-containing antineoplastic drug bortezomib, a proteasome inhibitor used for the treatment of multiple myeloma—has further sparked interest in boron compounds and their mechanism of action [18].

As an example, the boron derivative halogenated boroxine was suggested to inhibit the enzyme catalase, thus leading to higher production of hydrogen peroxide [75], as well as carbonic anhydrases in cancer cells [76]. However, the synthesis and the applications of novel boron derivatives have been well summarized in some recent reviews [17,18,77].

## 6. Conclusions and Future Directions

In conclusion, data from the literature suggest that BA could modulate proliferation and death in cancer cells. However, the huge differences in the concentrations used and the different sensitivity of the cancer cell lines to BA suggest that further in vivo experiments are required both to identify cancer types where BA could be really effective at concentrations realistically achievable in the tumor microenvironment, and to evaluate the possible use of BA in combination with chemotherapeutics or ferroptosis inducers. The development of derivatives capable of targeting tumor cells in a more specific way could obviously make the anti-tumor action of boron more effective.

## Data Availability

All data is contained within the article.
